# Evaluating the Impact of Maternal Lipid Profiles on Fetal Cardiac Function at Mid-Gestation: An Observational Study

**DOI:** 10.3390/clinpract14060204

**Published:** 2024-11-27

**Authors:** Biliana Belovan, Zoran Laurentiu Popa, Cosmin Citu, Ioana Mihaela Citu, Ioan Sas, Adrian Ratiu

**Affiliations:** 1 Doctoral School, “Victor Babes” University of Medicine and Pharmacy, Eftimie Murgu Square 2, 300041 Timisoara, Romania; belovan.biliana@umft.ro; 2 Department of Obstetrics and Gynecology, “Victor Babes” University of Medicine and Pharmacy, Eftimie Murgu Square 2, 300041 Timisoara, Romania; citu.ioan@umft.ro (C.C.); sas.ioan@umft.ro (I.S.); ratiu.adrian@umft.ro (A.R.); 3 Department of Internal Medicine I, “Victor Babes” University of Medicine and Pharmacy, Eftimie Murgu Square 2, 300041 Timisoara, Romania; citu.ioana@umft.ro

**Keywords:** maternal dyslipidemia, fetal cardiac function, echocardiography, pregnancy, lipid profiles

## Abstract

**Background:** Maternal dyslipidemia during pregnancy may influence fetal cardiac development and function, potentially predisposing offspring to cardiovascular diseases later in life. This study aims to evaluate the relationship between maternal lipid profiles and fetal cardiac function at mid-gestation, utilizing detailed echocardiographic assessments. **Methods:** In this prospective cohort study conducted at the Obstetrics and Gynecology Clinic of the Timișoara Municipal Emergency Hospital, 19 pregnant women aged 27–40 years were recruited and divided into two groups based on their triglyceride levels: Group A (triglycerides ≤ 150 mg/dL, *n* = 48) and Group B (triglycerides > 150 mg/dL, *n =* 28). Maternal demographic data and lipid profiles were recorded. Fetal echocardiographic measurements, including global longitudinal strain and ventricular function parameters, were obtained between 20 and 24 weeks of gestation. Statistical analyses, including subgroup comparisons, correlations, and regression analyses, were performed. **Results:** Maternal BMI was significantly higher in Group B compared to Group A (31.94 ± 2.80 vs. 27.01 ± 2.40 kg/m^2^, *p* < 0.001). Group B showed higher mean triglyceride levels (163.43 ± 11.34 mg/dL) compared to Group A (131.42 ± 10.57 mg/dL, *p* < 0.001). Fetal echocardiographic measurements indicated reduced global longitudinal strain in fetuses of Group B mothers (LV strain: −19.86% ± 6.83% vs. −26.14% ± 5.92%, *p* = 0.017). Significant correlations were found between maternal triglyceride levels and fetal LV strain (*r =* 0.536, *p* = 0.019). Regression analysis identified maternal triglyceride levels and BMI as significant predictors of reduced fetal LV strain (β = 0.45, *p* = 0.021 and β = 0.39, *p* = 0.038, respectively). **Conclusions:** Elevated maternal triglyceride levels, LDL cholesterol, and BMI are associated with altered fetal cardiac function parameters at mid-gestation, suggesting that maternal lipid profiles may impact fetal cardiac development. These findings underscore the importance of monitoring lipid levels during pregnancy and suggest potential benefits of managing dyslipidemia to improve fetal cardiac outcomes. However, the study included only a small sample; therefore, the study needs to be continued with a larger group.

## 1. Introduction

Cardiovascular diseases (CVD) are the leading cause of mortality worldwide, accounting for an estimated 17.9 million deaths annually [1]. The concept of developmental origins of health and disease (DOHaD) posits that environmental factors during critical periods of fetal development can have long-term effects on health outcomes [2]. Maternal metabolic status, including lipid profiles during pregnancy, plays a crucial role in fetal development and may influence the risk of offspring developing CVD later in life [3,4].

Pregnancy induces significant physiological changes in lipid metabolism, characterized by increased levels of triglycerides and cholesterol, particularly in the third trimester [5]. While these changes are considered normal adaptations to meet the energy demands of the growing fetus, excessive elevations can lead to dyslipidemia, which has been associated with adverse pregnancy outcomes such as preeclampsia, gestational diabetes mellitus, and preterm birth [6,7].

Emerging evidence suggests that maternal dyslipidemia may impact fetal cardiac development and function [8,9]. Animal studies have demonstrated that maternal hypercholesterolemia can lead to atherosclerotic lesions in fetal arteries [10]. In humans, elevated maternal lipid levels have been linked to changes in fetal vascular function and structure [11]. However, the relationship between maternal lipid profiles and fetal cardiac function remains underexplored.

Advancements in fetal echocardiography have enabled the assessment of fetal cardiac function in utero, including measurements of global longitudinal strain (GLS), fractional area change (FAC), and ejection fraction (EF) [12,13]. GLS is a sensitive parameter that reflects myocardial deformation and has been used to detect subtle cardiac dysfunctions [14]. These non-invasive techniques provide valuable insights into the impact of maternal factors on fetal cardiac health.

Understanding the influence of maternal lipid profiles on fetal cardiac function is essential for developing strategies to optimize maternal and fetal health. Identifying modifiable maternal risk factors during pregnancy could lead to interventions that reduce the risk of CVD in offspring. This study aims to evaluate the impact of maternal dyslipidemia, specifically elevated triglyceride levels, on fetal cardiac function at mid-gestation. We hypothesize that maternal dyslipidemia is associated with altered fetal cardiac function parameters, which could serve as early indicators of cardiovascular risk.

By conducting detailed echocardiographic assessments and analyzing the relationship between maternal lipid profiles and fetal cardiac function, this study aims to evaluate the relationship between maternal lipid profiles and fetal cardiac function at mid-gestation, utilizing detailed echocardiographic assessments. The findings may have implications for prenatal care practices and highlight the importance of managing maternal lipid levels during pregnancy.

## 2. Materials and Methods

### 2.1. Legal and Ethical Considerations

The current study was designed as a single-center, prospective study at the Obstetrics and Gynecology Clinic of the Timisoara Municipal Emergency Hospital, affiliated with the Victor Babes University of Medicine and Pharmacy in Timisoara, Romania, during the period January 2023–January 2024. This observational study secured ethical approval from the Institutional Review Board at the Obstetrics and Gynecology Clinic of the Timisoara Municipal Emergency Hospital, adhering to the principles set out in the Declaration of Helsinki. Additionally, the study complies with the EU Good Clinical Practice Directive (2005/28/EC) and the guidelines provided by the International Council for Harmonization of Technical Requirements for Pharmaceuticals for Human Use (ICH), which emphasize informed consent, scientific validity, and the safeguarding of participants’ health and rights.

In alignment with the General Data Protection Regulation (GDPR) and relevant national data protection laws, our study incorporates stringent measures to protect personal data. All patient information was anonymized before analysis, effectively removing any identifiers that could be traced back to individuals. All patients included in the study provided informed consent for data acquisition, dissemination, and publication of research studies.

### 2.2. Inclusion Criteria and Study Groups

For this study, the inclusion criteria focused on selecting pregnant women between the ages of 27 and 40 years with singleton pregnancies, ensuring a homogeneous sample for assessing maternal and fetal outcomes. Eligibility was established at the first antenatal visit, which occurred between 8 and 14 weeks of gestation, to capture participants early in their pregnancy. This specific timeframe allowed for early interventions and monitoring, while also ensuring that all participants were at a comparable stage of fetal development at the start of the study. Only singleton pregnancies were included, thereby excluding multiple gestations such as twins or triplets to avoid confounding factors associated with multifetal pregnancies. All fetuses included were appropriate for gestational age.

The exclusion criteria were designed to eliminate confounding factors that could influence maternal or fetal outcomes, especially those unrelated to pregnancy or that could severely affect pregnancy progression. Women with pre-existing cardiovascular diseases, including chronic hypertension or heart failure, were excluded due to the high risk these conditions pose to both the mother and fetus. Similarly, participants with a history of pre-existing diabetes mellitus (either type 1 or type 2) were excluded to avoid complications arising from glycemic variability. Preterm births before 36 weeks were excluded. Patients with high cholesterol were excluded, allowing the selection of only those with and without high triglyceride levels. Chronic kidney disease was also an exclusion criterion, as it can significantly impact pregnancy outcomes and could have complicated the interpretation of results. We also excluded fetuses that were small for gestational age or those with fetal growth restriction. Lastly, pregnancies with known fetal congenital anomalies or chromosomal abnormalities detected during prenatal screenings were excluded, as these factors can independently influence fetal development and maternal health. Additionally, severe systemic diseases unrelated to pregnancy were grounds for exclusion to focus on pregnancy-specific health issues without the interference of other significant health complications. We also excluded pregnant women with gestational hypertension and thyroid disease to avoid confounding effects.

### 2.3. Study Variables

At enrollment, maternal demographic data, including age, height, and weight, were recorded, and Body Mass Index (BMI) was calculated using the formula BMI = weight (kg)/[height (m)]^2^. Maternal weight was also measured at each antenatal visit. Fasting lipid profiles were collected during the second trimester (20–24 weeks of gestation), which included measurements of total cholesterol, low-density lipoprotein (LDL) cholesterol, high-density lipoprotein (HDL) cholesterol, and triglycerides. Blood samples for lipid analysis were obtained following an overnight fast of at least 8 h and were processed using standardized enzymatic methods.

### 2.4. Fetal Echocardiographic Assessments

Fetal echocardiographic examinations were conducted between 20 and 24 weeks of gestation using a Voluson E10 ultrasound machine (GE Healthcare, Chicago, IL, USA), equipped with a 4–8 MHz transducer. These examinations were performed by a single experienced operator who was blinded to the maternal lipid profiles to ensure unbiased assessment. The key measurements included global longitudinal strain (GLS) of both the left ventricle (LV) and right ventricle (RV) using speckle-tracking echocardiography, and fractional area change (FAC) for both ventricles, calculated as [(end-diastolic area − end-systolic area)/end-diastolic area] × 100%. Ejection fraction (EF) was determined using the modified Simpson’s method, while stroke volume (SV) and cardiac output (CO) were calculated based on ventricular dimensions and heart rate. Additional parameters assessed included ventricular sizes, end-diastolic diameters, fractional shortening, and sphericity indices, providing a comprehensive evaluation of fetal cardiac function.

### 2.5. Birth Outcomes

Birth outcomes were documented, including gestational age at delivery, mode of delivery (either cesarean section or vaginal birth), birth weight, and Apgar scores at both 1 and 5 min. These metrics provided essential information on the timing and type of delivery, as well as the newborn’s immediate health status following birth.

### 2.6. Group Classification

Participants were divided into two groups based on maternal triglyceride levels measured during the second trimester: Group A (Control Group): Triglycerides ≤ 150 mg/dL (*n =* 48); Group B (Dyslipidemia Group): Triglycerides > 150 mg/dL (*n =* 28). This cutoff was selected based on the American College of Obstetricians and Gynecologists (ACOG) guidelines, which consider triglyceride levels above 150 mg/dL as elevated during pregnancy [15].

### 2.7. Statistical Analysis

To determine the appropriate sample size for this study, we utilized the standard formula for comparing two independent means: *n =* [(Zα/2 + Zβ)^2^ × (2σ^2^)]/Δ^2^. In this formula, n represents the sample size, Zα/2 is the z-score corresponding to the desired significance level (α = 0.05, Zα/2 = 1.96), Zβ is the z-score corresponding to the desired power (80%, Zβ = 0.84), σ is the standard deviation of the outcome measure, and Δ is the expected difference between the two groups (effect size). Assuming a moderate effect size (Cohen’s d = 0.5), a significance level of 0.05, and a power of 80%, the calculation yielded an estimated sample size of approximately 64 participants (32 per group). To account for potential dropouts, the target sample size was increased by 10–15%, resulting in a final goal of 72–75 participants. Ultimately, the study successfully recruited 76 participants (48 in Group A and 28 in Group B), ensuring adequate statistical power to detect significant associations between maternal lipid profiles and fetal cardiac function.

Data were analyzed using SPSS Statistics version 25.0 (IBM Corp., Armonk, NY, USA). Continuous variables were expressed as mean ± standard deviation (SD), and categorical variables were presented as frequencies and percentages. Comparisons between groups were performed using the independent samples *t*-test for normally distributed continuous variables and the Mann–Whitney U test for non-normally distributed data. The Chi-square test or Fisher’s exact test was used for categorical variables. Pearson correlation coefficients were calculated to assess the relationships between maternal lipid levels and fetal echocardiographic parameters. Multiple linear regression analyses were conducted to identify predictors of fetal cardiac function parameters, adjusting for potential confounders such as maternal age and BMI. A *p*-value of less than 0.05 was considered statistically significant. All tests were two-tailed.

## 3. Results

Maternal characteristics such as age and height were analyzed, revealing no statistically significant differences between the groups, with maternal ages averaging 34.67 ± 4.12 years in Group A and 34.29 ± 4.07 years in Group B (*p* = 0.806), and heights of 164.00 ± 5.35 cm in Group A compared to 167.14 ± 4.88 cm in Group B (*p* = 0.239). However, notable differences were observed in weight and Body Mass Index (BMI), with Group A averaging 72.25 ± 12.26 kg and a BMI of 27.01 ± 2.40 kg/m^2^, while Group B exhibited higher averages of 89.43 ± 9.17 kg and 31.94 ± 2.80 kg/m^2^, respectively, with significant *p*-values of 0.003 and <0.001, as presented in Table 1.

Table 2 details maternal lipid profiles, showing significantly elevated total cholesterol levels of 137.75 ± 8.58 mg/dL in Group A versus 162.29 ± 11.80 mg/dL in Group B, and LDL cholesterol levels of 119.83 ± 8.67 mg/dL compared to 146.14 ± 13.38 mg/dL, both with *p*-values < 0.001. Triglycerides also varied significantly between the groups, with Group A at 131.42 ± 10.57 mg/dL and Group B at 163.43 ± 11.34 mg/dL (*p* < 0.001). These adverse lipid profiles in Group B were contrasted by non-significantly different HDL cholesterol levels, which averaged 40.08 ± 2.96 mg/dL in Group A and 37.94 ± 2.10 mg/dL in Group B (*p* = 0.091).

Fetal echocardiographic measurements, presented in Table 3, showed significant differences in LV global longitudinal strain, with Group A recording −26.14 ± 5.92% compared to −19.86 ± 6.83% in Group B (*p* = 0.017). Similar trends were observed in LV fractional area change (47.83 ± 9.68% in Group A vs. 38.71 ± 10.60% in Group B, *p* = 0.041) and ejection fraction (61.96 ± 6.12% in Group A vs. 55.14 ± 6.82% in Group B, *p* = 0.031). These results suggested poorer fetal cardiac function in Group B, potentially linked to maternal health profiles, particularly those related to obesity and lipid levels.

Correlation analyses, presented in Table 4, demonstrated significant associations between fetal LV global longitudinal strain and maternal triglycerides (*r =* 0.536, *p* = 0.019), LDL cholesterol (*r =* 0.493, *p* = 0.033), and BMI (*r =* 0.57, *p* = 0.012). The negative correlation with HDL cholesterol (*r =* −0.376) was not significant (*p* = 0.116), indicating that higher maternal triglycerides, LDL cholesterol, and BMI are linked with decreased fetal cardiac functionality.

Multiple linear regression analysis, shown in Table 5, revealed that each unit increase in maternal triglycerides resulted in a 0.45 increase in fetal LV strain (*p* = 0.021), and each unit increase in BMI led to a 0.39 increase (*p* = 0.038). Maternal age did not significantly affect fetal LV strain, indicated by a coefficient of −0.05 (*p* = 0.795), emphasizing the stronger impact of maternal metabolic factors compared to age.

Table 6 presents an assessment of birth outcomes, showing no significant differences in gestational age at birth (37.75 ± 4.00 weeks in Group A vs. 37.86 ± 2.12 weeks in Group B, *p* = 0.937), birth weight (3398 ± 358 g in Group A vs. 3252 ± 460 g in Group B, *p* = 0.493), or Apgar scores at 1 and 5 min (8.58 ± 0.67 vs. 8.29 ± 0.76, *p* = 0.334 and 9.25 ± 0.45 vs. 9.14 ± 0.38, *p* = 0.592, respectively). The similarity in immediate birth outcomes across both groups suggested that the maternal and fetal differences observed did not translate into variations in neonatal well-being at birth.

The data presented in Table 7 suggest a clear trend in which increasing maternal BMI is associated with worsening fetal cardiac function. Specifically, the LV global longitudinal strain showed significant deterioration as BMI categories rose from normal to obese, moving from an average of −25.84% in normal-weight mothers to −18.92% in obese mothers (*p* = 0.017). This pattern suggests that higher maternal BMI may negatively impact fetal cardiac contractility. Similarly, the LV fractional area change, which reflects the percentage change in ventricular area during the cardiac cycle, decreased significantly with higher BMI, from 48.29% in the normal group to 39.84% in the obese group (*p* = 0.043).

The analysis of fetal cardiac outcomes in relation to maternal triglyceride levels as shown in Table 8 reveals a notable decline in fetal cardiac health with increasing triglyceride concentrations. Specifically, the LV global longitudinal strain deteriorated significantly as triglyceride levels moved from below 150 mg/dL to above 300 mg/dL, with a strain decrease from −26.34% to −16.89% (*p* = 0.008). This pattern indicates that higher maternal triglycerides are associated with reduced fetal cardiac contractility, a critical marker of cardiac health. Similarly, the LV fractional area change, a measure of the heart’s ability to change area during contraction and relaxation cycles, also showed a significant decrease across the triglyceride categories, from 49.13% in the lowest category to 36.87% in the highest (*p* = 0.015).

## 4. Discussion

### 4.1. Analysis of Findings

This observational study investigated the impact of maternal dyslipidemia on fetal cardiac function at mid-gestation. The key findings indicate that elevated maternal triglyceride levels and BMI are associated with altered fetal cardiac function, evidenced by reduced LV global longitudinal strain, fractional area change, and ejection fraction. Fetuses of mothers with elevated triglyceride levels showed significantly reduced LV strain compared to those of mothers with normal triglyceride levels. This suggests impaired myocardial deformation and potential subclinical cardiac dysfunction in fetuses exposed to maternal dyslipidemia.

The higher stroke volume and cardiac output observed in Group B, which included women with elevated triglycerides and BMI, can likely be attributed to maternal metabolic and hemodynamic changes associated with obesity and hypertriglyceridemia. Elevated BMI is often linked with increased blood volume and cardiac workload to meet the metabolic demands of the mother and fetus. This can lead to a compensatory increase in cardiac output and stroke volume as the heart pumps more blood per beat to ensure adequate oxygen and nutrient supply. Additionally, hypertriglyceridemia can contribute to increased vascular resistance, which further elevates the heart’s workload. These hemodynamic adaptations, while initially compensatory, may also reflect early subclinical signs of altered cardiac function in the fetus, as seen with reduced cardiac strain and fractional area change in Group B.

Maternal dyslipidemia during pregnancy has been associated with adverse outcomes, including preeclampsia, gestational diabetes, and preterm birth [16]. However, its impact on fetal cardiac function has not been extensively studied. Our findings align with previous research indicating that maternal metabolic abnormalities can affect fetal cardiac development [17]. For example, one study found that maternal dyslipidemia was associated with alterations in fetal cardiac structure and function [18].

Reduced LV strain in fetuses of dyslipidemic mothers may reflect changes in myocardial architecture or function due to exposure to elevated lipid levels. Potential mechanisms include lipid infiltration, oxidative stress, and inflammatory processes affecting the developing myocardium [19]. The positive correlation between maternal triglyceride levels and fetal LV strain found in our study supports the notion that higher maternal lipid levels are directly related to impaired fetal cardiac function. Similarly, maternal BMI was a significant predictor of reduced fetal LV strain, suggesting that maternal obesity contributes to fetal cardiac alterations.

In examining fetal cardiac function, the study by Domínguez-Gallardo et al. [20] highlighted significant differences in left ventricular (LV) strain between fetuses diagnosed with fetal growth restriction (FGR) and those appropriate for gestational age (AGA). The longitudinal cohort design revealed that FGR fetuses exhibited consistently lower global and segmental LV longitudinal strain values throughout gestation, indicating subclinical cardiac dysfunction potentially due to placental insufficiency. In a similar manner, the study by Ishii et al. [21] provided baseline measurements of ventricular strain in a healthy fetal population, documenting average mid-ventricular circumferential strain (LVCS) at 18.7 ± 3.3 and longitudinal strain (LVLS) at 15.2 ± 2.7. These values did not correlate with gestational age, suggesting stable cardiac function in the absence of complications. Notably, while Ishii et al. established normal reference ranges for fetal cardiac strain, Domínguez-Gallardo et al. demonstrated how deviations from these norms could be indicative of underlying health issues like FGR.

Similarly, the study by Änghagen et al. [22] explored the developmental differences in left ventricular strain between infants with intrauterine growth restriction (IUGR) and healthy controls over the first three months of life. Initially, at birth, LVLS measurements did not significantly differ between the IUGR group [−15.76 (3.12)%] and controls [−15.53 (3.56)%]. However, by 3–4 months, the control group demonstrated a significant increase in LVLS to [−20.91 (3.31)%], unlike the IUGR group, which showed a smaller change to [−17.80 (3.82)%], suggesting a persistent impairment in cardiac function among the IUGR infants. In a similar manner, the study by Ozawa et al. [23] assessed the cardiovascular impact on fetal sheep supported by the EXTrauterine Environment for Neonatal Development (EXTEND) system, observing the heart rate, mean arterial pressure, and ventricular strain over three weeks. While, initially, both right and left ventricular strain values decreased, indicating a temporary decline in contractility, they returned to baseline by the third week, suggesting a period of physiological adjustment to the artificial environment.

In a similar manner, Domínguez-Gallardo et al. [24] utilized two-dimensional speckle-tracking echocardiography to scrutinize left ventricular function across various severity stages of fetal growth restriction (FGR), revealing that small for gestational age (SGA) and Stage I FGR fetuses displayed higher global longitudinal strain levels, with measurements being −15.76 ± 3.12% in SGA and similar in Stage I, compared to −17.80 ± 3.82% in more severe Stage ≥ II FGR cases. This progression indicates deteriorating cardiac function as FGR severity increases. Similarly, Dargahpour Barough et al.’s [25] study used feature tracking magnetic resonance imaging (FT-MRI) to assess myocardial strain in fetuses. They reported median global longitudinal strain values of −13.2% in fetuses with congenital heart disease (CHD) versus −18.9% in healthy controls, highlighting significant differences in cardiac function.

In the study by van Oostrum et al. [26], two-dimensional speckle-tracking echocardiography (2D-STE) was used to generate reference values for fetal myocardial deformation in 124 healthy fetuses, capturing a total of 592 left ventricular and 566 right ventricular global longitudinal strain and strain rate (GLSR) measurements. This extensive dataset established that right ventricular GLS values were significantly higher (less negative) compared to left ventricular values across all gestational ages, indicating a developmental disparity between the two ventricles. In a similar manner, the study by Yovera et al. [27] assessed the impact of gestational diabetes mellitus (GDM) on fetal cardiac function and morphology, involving 112 women with GDM and 224 control subjects. Their findings highlighted that fetuses from GDM pregnancies exhibited a consistent reduction in right ventricular GLS compared to controls, with adjustments showing mean differences of 0.7% (24 + 0 to 32 + 0 weeks) and 0.9% (32 + 1 to 40 + 1 weeks).

Nevertheless, it is important to take into account that Romania’s cesarean section (C-section) rate is notably higher than the European Union (EU) average. In 2017, Romania reported a C-section rate of 44.1%, the second highest in the EU, surpassed only by Cyprus at 54.8% [28]. This figure significantly exceeds the EU average, which was approximately 25% in the same year. Several factors contribute to this disparity. A study conducted in a Romanian tertiary maternity hospital revealed that many women prefer C-sections due to perceptions of safety and convenience, even in the absence of medical indications [29]. Additionally, healthcare system characteristics, such as the availability of medical resources and the influence of private healthcare services, may encourage elective C-sections. These elements, combined with cultural attitudes towards childbirth, contribute to Romania’s elevated C-section rate compared to the EU average.

While it is true that preterm birth can independently influence cardiac function [30], our study specifically aimed to isolate the impact of maternal lipid profiles on fetal cardiac development. We accounted for potential confounders by excluding participants with known conditions that can lead to preterm delivery, such as gestational hypertension, pre-existing cardiovascular diseases, or diabetes. Additionally, the correlation analyses demonstrated significant associations between elevated maternal triglyceride levels and altered fetal cardiac function, independent of gestational age at delivery. However, we acknowledge that the interplay between preterm birth and maternal lipid levels could be complex, and further research with larger sample sizes would help clarify these interactions.

These findings underscore the importance of monitoring maternal lipid profiles and BMI during pregnancy. Identifying and managing dyslipidemia could have implications for fetal cardiac health and potentially reduce the risk of cardiovascular diseases later in life. Therefore, interventions such as dietary modifications, physical activity, and, in some cases, pharmacotherapy may be considered to manage dyslipidemia during pregnancy, with careful consideration of fetal safety.

### 4.2. Study Limitations

The study’s small sample size limits its statistical power and generalizability, and its observational design prevents establishing causality. Additionally, potential confounders, despite adjustments in regression analyses, may have influenced the results. Also, there was no comparison to the late-term and after-birth stages due to resources limitations and potential loss to follow-up that would further reduce the already small sample size. Future research should focus on larger sample sizes with longitudinal follow-up to confirm these findings and investigate the long-term implications of altered fetal cardiac function due to maternal dyslipidemia. Moreover, exploring interventions to manage maternal lipid levels during pregnancy and their effects on fetal outcomes is warranted.

## 5. Conclusions

Maternal hypertriglyceridemia, elevated LDL cholesterol levels, and increased BMI may be associated with changes in fetal cardiac function at mid-gestation between 20–24 weeks. However, given the limitations of this study’s small sample size, these findings should be interpreted with caution. While the results highlight a potential link between maternal lipid profiles and fetal cardiac development, further randomized studies involving larger population groups are needed to confirm these associations and better understand their clinical implications. This study’s findings underscore the importance of monitoring and managing dyslipidemia during pregnancy, but more robust evidence is required before making definitive clinical recommendations.

## Figures and Tables

**Table 1:** Maternal characteristics by group.

Variables (Mean ± SD)	Group A (*n* = 48)	Group B (*n =* 28)	*p*-Value
Maternal age, years	34.67 ± 4.12	34.29 ± 4.07	0.806
Height (cm)	164.00 ± 5.35	167.14 ± 4.88	0.239
Weight (kg)	72.25 ± 12.26	89.43 ± 9.17	0.003
BMI (kg/m^2^)	27.01 ± 2.40	31.94 ± 2.80	<0.001

BMI—Body Mass Index; SD—Standard Deviation.

**Table 2:** Maternal lipid profiles by group.

Variables (Mean ± SD)	Group A (*n* = 48)	Group B (*n* = 28)	*p*-Value
Total Cholesterol (mg/dL)	137.75 ± 8.58	162.29 ± 11.80	<0.001
LDL Cholesterol (mg/dL)	119.83 ± 8.67	146.14 ± 13.38	<0.001
HDL Cholesterol (mg/dL)	40.08 ± 2.96	37.94 ± 2.10	0.091
Triglycerides (mg/dL)	131.42 ± 10.57	163.43 ± 11.34	<0.001

SD—Standard Deviation; HDL—High-Density Lipoprotein; LDL—Low-Density Lipoprotein; TNF—Tumor Necrosis Factor.

**Table 3:** Fetal echocardiographic measurements by group.

Variables (Mean ± SD)	Group A (*n* = 48)	Group B (*n* = 28)	*p*-Value
Gestational Age at Assessment (weeks)	22.75 ± 1.82	22.86 ± 1.86	0.882
LV Global Longitudinal Strain (%)	–26.14 ± 5.92	–19.86 ± 6.83	0.017
RV Global Longitudinal Strain (%)	–23.17 ± 6.52	–17.71 ± 6.08	0.064
LV Fractional Area Change (%)	47.83 ± 9.68	38.71 ± 10.60	0.041
RV Fractional Area Change (%)	45.58 ± 9.27	38.14 ± 9.91	0.090
Ejection Fraction (%)	61.96 ± 6.12	55.14 ± 6.82	0.031
Stroke Volume (mL)	0.53 ± 0.22	0.96 ± 0.62	0.049
Cardiac Output (mL/min)	56.18 ± 16.80	106.24 ± 72.37	0.038

SD—Standard Deviation; RV—Right Ventricle; LV—Left Ventricle.

**Table 4:** Correlations between maternal lipid levels and fetal LV global longitudinal strain.

Maternal Variable	Correlation Coefficient (r)	*p*-Value
Triglycerides	0.536	0.019
LDL Cholesterol	0.493	0.033
HDL Cholesterol	−0.376	0.116
BMI	0.57	0.012

HDL—High-Density Lipoprotein; LDL—Low-Density Lipoprotein; BMI—Body Mass Index.

**Table 5:** Multiple linear regression analysis predicting fetal LV global longitudinal strain.

Variables	Unstandardized Coefficient (β)	Standard Error	*p*-Value
Triglycerides	0.45	0.17	0.021
BMI	0.39	0.17	0.038
Maternal Age	–0.05	0.19	0.795
(Constant)	–41.28	7.8	<0.001

BMI—Body Mass Index.

**Table 6:** Birth outcomes by group.

Variables	Group A (*n* = 48)	Group B (*n* = 28)	*p*-Value
Gestational Age at Birth (weeks)	37.75 ± 4.00	37.86 ± 2.12	0.937
Preterm proportions (*n*, %)	10 (20.8%)	9 (32.1%)	0.297
Birth Weight (g)	3398 ± 358	3252 ± 460	0.493
Apgar Score at 1 min	8.58 ± 0.67	8.29 ± 0.76	0.334
Apgar Score at 5 min	9.25 ± 0.45	9.14 ± 0.38	0.592
Cesarean Delivery	36 (75.0%)	24 (85.7%)	0.608

**Table 7:** Fetal cardiac outcomes by maternal BMI category.

Maternal BMI Category	*n*	LV Global Longitudinal Strain (%)	LV Fractional Area Change (%)	*p*-Value (LV GLS)	*p*-Value (LV FAC)
Normal (18.5–24.99)	40	−25.84 ± 4.98	48.29 ± 8.47	-	-
Overweight (25–29.99)	24	−23.67 ± 5.23	45.03 ± 7.92	0.276	0.157
Obese (≥30)	12	−18.92 ± 6.04	39.84 ± 9.36	0.017	0.043

BMI—Body Mass Index; LV—Left Ventricle; GLS—Global Longitudinal Strain; FAC—Fractional Area Change.

**Table 8:** Fetal cardiac outcomes by maternal triglyceride levels.

Maternal Triglyceride Levels (mg/dL)	*n*	LV Global Longitudinal Strain (%)	LV Fractional Area Change (%)	*p*-Value (LV GLS)	*p*-Value (LV FAC)
<150	48	−26.34 ± 5.12	49.13 ± 8.23	-	-
150–300	17	−22.47 ± 6.19	43.79 ± 8.76	0.036	0.022
>300	11	−16.89 ± 7.41	36.87 ± 10.03	0.008	0.015

BMI—Body Mass Index; GLS—Global Longitudinal Strain; FAC—Fractional Area Change.

## Data Availability

The data presented in this study are available on request from the corresponding author.
